# Aging and Longevity: Why Knowing the Difference Is Important to Nutrition Research

**DOI:** 10.3390/nu3030274

**Published:** 2011-02-28

**Authors:** Roger B. McDonald, Rodney C. Ruhe

**Affiliations:** Department of Nutrition, University of California, One Shields Ave, Davis, CA 95616, USA; Email: rcruhe@ucdavis.edu

**Keywords:** chance and aging, evolution of longevity, genetic determinism, nutrition recommendations

## Abstract

Life expectancies after the age of 70 and the number of individuals living with age-related chronic conditions that affect daily activities continue to increase. Age-specific nutritional recommendations may help to decrease the incidence or severity of age-related debilitating chronic disorders. However, research in this area has seen limited success in identifying nutrition-related mechanisms that underlie the functional loss and chronic conditions that occur as a function of time. We believe that the limited success in establishing age-specific nutrition recommendations for the older population reflects, at least in part, research designs that fail to consider the evolutionary and biological bases of aging and longevity. Longevity has evolved as a by-product of genes selected for their contribution in helping the organism survive to the age of reproduction. As such, the principle of genetic determinism provides an appropriate underlying theory for research designs evaluating nutritional factors involved with life span. Aging is not a product of evolution and reflects stochastic and/or random events that most likely begin during the early, reproductively-active years. The genetic determinism model by which young (normal, control) are compared to old (abnormal, experimental) groups will not be effective in identifying underlying mechanisms and nutritional factors that impact aging. The purpose of this commentary is to briefly discuss the difference between aging and longevity and why knowing the difference is important to nutrition research and to establishing the most precise nutritional recommendations possible for the older population.

## 1. Introduction

Life expectancies after the age of 70 and especially after the age of 85 have yet to reach a plateau. While life expectancy after the age of 70 continues to increase, so too do the number of individuals living with age-related chronic conditions that affect daily activities. Many believe that age-specific nutritional recommendations for the older population may help to decrease the incidence or severity of debilitating, non-disease disorders that occur with increasing age. However, research in this area has seen limited success in identifying nutrition-related mechanisms that underlie the functional loss and chronic conditions that occur as a function of time.

We suggest that lack of success in establishing age-specific nutritional recommendations for the older population reflects, at least in part, research designs that do not consider the biological differences between aging and longevity. Failure to consider these differences may also lead to difficulty in precisely defining aging or longevity, a prerequisite to efficient experimental designs. Distinguishing human biological aging from longevity can be difficult due to the fact that the rate of aging may affect the length of the life span. Moreover, the lack of predation, control of childhood diseases, and effective therapies for life-threatening disease have extended the human post-reproductive life span, making the distinction between aging and longevity even more difficult. Nonetheless, such a distinction will be necessary to develop the most effective research designs evaluating the mechanisms that underlie the impact of nutrition on aging or longevity. Although we acknowledge that there are many valid definitions for aging, we suggest that biological aging be defined as the progressive, event-dependent decline in the ability to maintain biochemical/physiological function. Longevity is the length of the life span independent of the biological aging process. This brief commentary will provide justification for these two definitions. 

The ability to define aging and longevity separately has become possible only in recent years. Biogerontological research conducted during the last two decades has, to a large degree, solved the evolutionary problem of longevity and aging. Evolutionary theorists have mathematically and empirically demonstrated that longevity is genetically determined from genes that are selected for a reproductive advantage [1,2]. As such, the principle of genetic determinism provides an appropriate underlying theory for research designs evaluating nutritional factors involved with life span. Investigations evaluating possible gene-nutrient interaction should prove valuable in the search for nutrients that affect longevity. On the other hand, aging is not a product of selective evolution. The aging process more closely reflects chance events that affect biological systems during development or during the early reproductively-active years [3]. The genetic determinism model by which young (normal, control) are compared to old (abnormal, experimental) groups will not be effective in identifying mechanisms by which nutritional factors affect aging. Aging will be best understood by evaluating biological systems during development that are most susceptible to time- or event-dependent alteration leading to functional loss and chronic conditions in old age. 

Why should a nutrition researcher know the difference between aging and longevity? In the next decade and a half in economically-developed countries, the population of individuals who will be over the age of 70 years will rise from the current 13% to almost 20%. In the United States that percent increase represents an additional 35 million people. Given the disproportionate amount of health care dollars spent on this population, much of which is covered by entitlement programs, we must do all we can to insure a healthy aging population—anything less could be economically catastrophic. Dietary recommendations are a significant part of the overall strategy for improving the health of the aged population. 

In an effort to improve the health of older Americans through dietary recommendations, the Committee on the Dietary Reference Intakes (CDRI) has added two age classifications, 51–70 and 70+. However, after 12 years of having these new age classifications, recommendations in the 51–70 and 70+ age groups do not, in general, differ significantly from younger adult age groups [4]. The CDRI attributes the lack of age-specific recommendations to insufficient research. While more research will be necessary, the inability to establish age-specific recommendations also reflects results from research designs that have based their hypotheses on outdated theory. We believe that precise and sustainable recommendations for the older population will come from research designs that consider the most up-to-date findings from biogerontology, including the related but distinct biological processes of aging and longevity [5,6,7]. Knowing the difference between aging and longevity will be fundamental to developing the strongest designs for nutrition research aimed at establishing recommendations and improving the health of the older population.

In this commentary, we discuss the difference between aging and longevity and why knowing this difference is important to nutrition research. To this end, a brief review will be presented of evolutionary and genetic findings showing that aging lacks genetic determinism whereas longevity evolved. Through this scientific evidence we will show why knowing the difference between aging and longevity is critically important to designing appropriate nutrition research that can be used for nutritional recommendations in the elderly population. A brief discussion will follow as to why establishing nutritional recommendations for the older population will be best achieved by first evaluating genetic/metabolic pathways in young populations that are most susceptible to the chance events leading to age-related functional loss. The interaction between aging and disease will not be covered here. Previous reviews have described thoroughly the differences between aging and disease and the reasons why using disease as a research model for aging may not be tenable [5,6].

## 2. Imprecise Terminology: Aging *vs.* Longevity

In 1952, Sir Peter Medawar delivered a lecture at the University of London focusing on the evolutionary problem of aging entitled “The Unsolved Problem of Biology” [8]. He argued that natural selection could not have worked to fix genes causing the loss in physiological function that begins as reproductive success declines. Medawar reasoned that the extrinsic hazards of the environment present throughout evolutionary history would result in an age distribution that favored a young, reproductively-active population. The younger population would have had greater fitness simply because there were more of them than the population of reproductively-active older individuals. The high rate of reproduction (fitness) in young *vs.* old age groups would result in traits important for survival to reproduction age being selected over alleles expressing proteins that insure the maintenance of physiological function well past reproduction age. Aging could not have evolved through natural selection. 

Medawar’s suggestion that aging did not arise from natural selection came at a time when genetic determinism through natural section was universally accepted as the only explanation for evolution. Therefore, a hypothesis that could explain, in term of genetic determinism, how alleles giving rise to aging could be selected in the absence of natural selection was needed. To this end, Medawar turned to genetic drift, a population genetics theory predicting that alleles in small populations can be fixed in the general population’s genome as a matter of chance. Medawar was building on the ideas of Haldane [9] that a small population of late-life reproducers carried genes conveying the aging phenotype. This small population could contribute to the establishment of an aging phenotype because nonlethal but physiologically detrimental genes expressed only in late life could arise in a population because they would not have had any effect on reproduction. Natural selection would have selected neither for nor against these genes. Medawar named his evolutionary aging theory mutation accumulation.

Medawar’s verbal postulations on aging provided the foundation for scores of researchers investigating the evolutionary, genetic, and biological basis of aging and longevity. However, his theory on aging contained a significant flaw that has been perpetuated in research designs for over 50 years. That is, he used the terms aging and longevity interchangeably even though his theoretical models focused exclusively on longevity. Medawar’s model for aging/longevity did not consider the possibility that life span and the functional loss characterizing aging could have arisen through different evolutionary/biological processes. Subsequent work found that longevity and aging are distinct biological events. 

**Figure 1:**
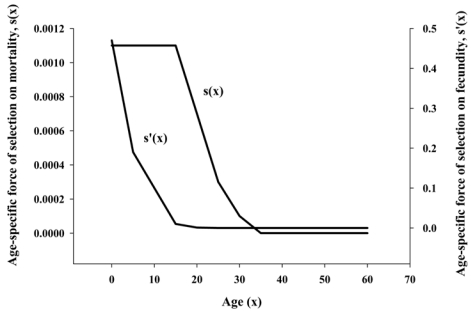
Hamilton’s calculation on the force of natural selection on mortality (*s*(*x*)) and fecundity (*s*’(*x*)). Data used are from life-tables of the United States between 2000 and 2004 (Adapted from [2]).

The differences between aging and longevity became clearer with the mathematical descriptions by W.D. Hamilton [1] and later by B. Charlesworth [10]. Hamilton’s mathematics agreed with Medawar’s verbal speculation that genes did indeed affect events leading to either aging and/or longevity. However, these genes were not the type that Medawar had suggested, *i.e.*, fixed by genetic drift and specific to the aging phenotype. Rather, Hamilton’s mathematical model predicted that longevity was determined by genes selected for reproductive success and not by genes that were expressed only late in life. His model showed that the force of natural selection on mortality was highest before the start of the reproduction phase and declined thereafter (Figure 1). Because the force of mortality was highest prior to reproduction, evolution would have worked to select genes that were necessary for combating mortality in early life, *i.e.*, surviving to reproduction age. Mortality (life span), therefore, must be related to genes selected for survival to reproduction age. Hamilton provided the first mathematically implicit evidence that life span (longevity) had evolved and was directly related to genes that optimized survival to the age of reproduction. Importantly, his model was specific to longevity and did not include variables of age-related functional loss. 

**Figure 2:**
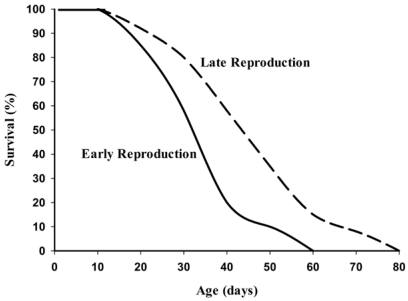
Life span of female *Drosophila* artificially selected for early and late life reproduction (Adapted with permission from John Wiley & Sons [12]).

Hamilton’s mathematical theory on longevity was a monumental breakthrough in the understanding of how life span, not aging, evolved. Based on his mathematical models, several laboratories using artificial selection methods to approximate evolution have supported Hamilton’s theories on the evolution of longevity [11,12,13]. Populations of *Drosophila* selected in the laboratory for their timing of reproduction showed that late-life reproducers do indeed live significantly longer than flies having their highest rate of reproduction early in the life span (Figure 2). Genetic studies in yeast, *C. elegans*, and *Drosophila* have also shown that genes affecting life span have been selected first and foremost for their role in enhancing survival to reproduction age (see review [14]). 

Mathematical models and empirical experimentation have unequivocally established that longevity evolved from genes selected for their impact on survival to the age of reproduction. Because these investigations did not include measures of age-related degeneration, conclusions as to the genetic basis of aging cannot be made. Nonetheless, hundreds if not thousands of publications exist describing research that either directly or indirectly implicate specific genes as being involved in age-related degeneration. Not one gene has been identified that causes osteoarthritis, presbyopia, sarcopenia, or any other of the hundreds of age-related degenerative and chronic disorders observed in the aged population. The reason for this is simple-these genes do not exist. 

## 3. The Disposable Nature of the Soma and the Cause of Aging

Aging arose serendipitously in evolutionary history as a result of a trade-off between the germ line and somatic cells in the distribution of resources. This trade-off has been developed into a formal theory by Thomas Kirkwood and is known as the Disposable Soma Theory of Aging [15]. The foundation of this theory lies directly in a basic principle of natural selection, *i.e.*, all environments have finite resources and individuals compete for these resources. Organisms that use the finite resources most efficiently will be the ones to successfully survive to reproduction age. The Disposable Soma Theory posits that the most efficient use of resources in multicellular organisms is to give highest priority to the cells that are responsible for the continuation of the species, *i.e.*, the cells of reproduction, or the germ line. Supporting cells, those of the soma, would only need enough resources to accomplish their primary task of supporting the germ line. That is, the soma could be disposed of once reproduction had occurred. 

But, where and how were those resources being spent? The Disposable Soma Theory predicts that that the distribution of resources by the early metazoans was preferentially diverted to repair mechanisms of the DNA in the germ line. This suggestion is consistent with the observation that the energetic cost for maintaining DNA fidelity is rather high. If, because of finite resources, an organism had to make an evolutionary choice between accuracy in the DNA of the germ cell or repair of a somatic cell, the germ cell would be chosen so as to provide the best chance of survival for the next generation. The immortality of the germ line has come at the cost of somatic mortality. 

## 4. Chance, Aging, and Nutritional Recommendation

The evidence presented thus far leads to the conclusion that aging is a random and/or stochastic phenomenon. A substantial literature exists describing that the primary mechanism associated with the random nature of aging is entropy, a property of the Second Law of Thermodynamics (see reviews [5,6,7]). Briefly, biological systems defend themselves against the unceasing disorder of entropy by continuously restoring the free energy lost to chemical reactions that maintain molecular fidelity (structure, function). At its very basic level, survival to reproduction age simply reflects the selection of genes that maintain free energy states conducive to life. However, there is no reproductive advantage for an individual to sustain molecular fidelity after the age of reproduction. Genes would not have been selected for the purpose of maintaining the high cost of combating entropy throughout the life span. The age-related decline in physiological function reflects a gradual loss in the ability to defend against the Second Law of Thermodynamics, *i.e.*, entropy. Importantly, the age-related loss in the ability to defend against entropy manifests purely as random events with respect to the physiological systems affected. 

The random nature of aging suggests that chance plays a significant part in determining the physiological system or systems that experience declining function in the older population. The role of chance as a factor in the aging process has been reviewed in detail [3]. Chance precludes genetic determinism and introduces an element of uncertainty that cannot be controlled easily in population-based research, the type of research commonly used as the basis for establishing nutritional recommendations. The CDRI recognizes the problem of random variation in the aging population when stating that research investigating dietary recommendations for the older population is confounded by increased error (random variation) in the data [4]. Moreover, the error becomes greater with advancing age, suggesting that nutritional recommendations based on the current scientific rationale, *i.e.*, genetic determinism, are even less accurate for the oldest age groups. 

Chance also raises the possibility that every sample population will be unique and that no sample population can be relied upon to provide meaningful predictive results for the entire aged population. Take for example the results from studies investigating the value of nutritional antioxidants as modulators of aging. Nutritional antioxidants prevent damage to the cell *in vitro* and the accumulation of cellular damage has found wide acceptance as a proximal cause of organism aging. The epidemiological data suggest that the rate of aging and age-related disease may decrease in populations consuming foods high in antioxidants [16,17]. Consistent with the epidemiological data, small sample size cross-sectional supplementation investigations tend to find positive outcomes between experimental (supplemented) and control (non-supplemented). However, several large randomized clinical trials have failed to demonstrate the benefit of antioxidant supplements in the aging population (see review [18]). That is, the small cross-sectional studies have not been reliable indicators of the usefulness of antioxidant nutrition in the general older population. 

The dominant role of chance in the aging process (but not longevity) leads to the inevitable conclusion that the fundamental process of biological aging cannot be modulated through interventions during old age. The nutrition researcher may find this statement difficult to accept given the overwhelming successes that nutrition interventions had on infant and childhood health. The reason that nutrition interventions improved the health of infants and children was that nutrients had targets to interact with, *i.e.*, genes selected for survival to reproduction age. Since aging did not arise through selective evolution, nutrients may or may not alter expression of genes in the older population. Recommendations for the older population arrived at by using genetic determinism will be unreliable—without genetic determinism, it is a matter of chance. 

If modulating aging by nutritional intervention during old age will not produce the desired results, how then will nutrition intervention be effective in improving the health of the older population? We suggest that the research approach should focus on possible variation in function among individuals in younger age groups that may predispose them to differential rates of functional decline during advancing age. We must focus our attention on the time in the life span in which genetic determinism has the greatest influence on random aging outcomes, *i.e.*, the development, juvenile, and reproductively-active periods. Only after gaining a clear understanding of how chance and randomness shape the genetic pathways in younger populations that, in turn, affect the outcomes in the older population, should nutrition recommendations be considered. 

Refocusing nutrition and aging research to be consistent with the current understanding of the cause of aging may not be as difficult as it first may appear. While a personal genomic approach will ultimately be required in humans, several investigative models exist that may prove useful. The Fetal Origins Hypothesis (a.k.a Fetal Basis of Adult Disease) suggests that some diseases have their bases *in utero* [19]. Most research in this area has focused on the relationship between fetal/maternal nutrition and adult-onset obesity. These investigations suggest that undernutrition *in utero* leads to higher incidences of adult-onset obesity, hypertension, cardiovascular disease, and type II diabetes [20]. Recently, a link between Parkinson’s and Alzheimer’s Disease and a toxic gestational environment during neurodevelopment has begun to emerge [21]. For example, the amyloidogenic proteins Aβ and Aβ precursor as well as BACE1 (the Aβ cleavage enzyme) are elevated in non-human primates exposed to lead during infancy [22]. Although the current focus of research linking the developmental environment with aging remains on age-related disease, it would not be surprising to find that the rate of aging and the trajectory in the age-related loss of soma maintenance, independent of overt disease, may also have fetal origins. 

The age-related alterations to the vascular system and the development of certain types of cancer have been used extensively as models of the aging phenotype. A substantial literature exists describing how dietary habits during childhood may induce dysfunction of various systems during aging. Some recent publications provide additional examples of specific research areas that should be given consideration [3,5,6] in this regard.

## 5. Conclusions

Evolutionary and genetic research has clearly established that longevity has evolved whereas aging is a random/stochastic process driven primarily by chance events occurring during development and the reproductive years. Knowing the difference between aging and longevity will determine, in part, the scientific approach to research questions aimed at evaluating the impact that nutrition may have on the aging population. If the primary purpose of the research is to determine the factors involved with longevity, then focusing on altering the expression of specific genes by specific nutrients will be appropriate. If, however, the aim of the research is to evaluate how nutritional interventions can modulate the aging process and improve the health of the older population, then the genetic determinism model will be inappropriate. Research designs that focus on chance events in young populations that lead to altered states of aging will be the more powerful. Effective nutritional recommendations for the aged population will most likely be ones that focus on dietary changes in the younger populations. 

## References

[1] Hamilton W.D. (1966). The moulding of senescence by natural selection. J. Theor. Biol..

[2] Rose M.R., Burke M.K., Shahrestani P., Mueller L.D. (2008). Evolution of ageing since Darwin. J. Genet..

[3] Finch C.E., Kirkwood T.B. (2000). Chance, Development, and Aging.

[4] Otten J.J., Hellwig J.P., Meyer L.D. (2006). DRI, Dietary Reference Intakes: Essential Guide to Nutrient Requirments.

[5] Hayflick L. (2007). Entropy explains aging, genetic determinism explains longevity, and undefined terminology explains misunderstanding both. PLoS Genet..

[6] Hayflick L. (2007). Biological aging is no longer an unsolved problem. Ann. N. Y. Acad. Sci..

[7] Lithgow G.J. (2006). Why aging isn’t regulated: A lamentation on the use of language in aging literature. Exp. Gerontol..

[8] Medawar P.B. (1952). An Unsolved Problem of Biology.

[9] Haldane J.B.S. (1942). New Paths in Genetics.

[10] Charlesworth B. (1970). Selection in populations with overlapping generations. I. The use of Malthusian parameters in population genetics. Theor. Popul. Biol..

[11] Luckinbill L.S., Arking R., Clare M.J., Cirocco W.C., Buck S.A. (1984). Selection for delayed senescence in *Drosophlia melanogaster*. Evolution.

[12] Rose M.R. (1984). Laboratory evolution of postponed senescence in Drosophila melanogaster. Evolution.

[13] Stearns S.C., Ackermann M., Doebeli M., Kaiser M. (2000). Experimental evolution of aging, growth, and reproduction in fruitflies. Proc. Natl. Acad. Sci. USA.

[14] Kenyon C. (2005). The plasticity of aging: Insights from long-lived mutants. Cell.

[15] Kirkwood T.B., Holliday R. (1979). The evolution of ageing and longevity. Proc. R. Soc. Lond. B Biol. Sci..

[16] Hirvonen T., Virtamo J., Korhonen P., Albanes D., Pietinen P. (2000). Intake of flavonoids, carotenoids, vitamins C and E, and risk of stroke in male smokers. Stroke.

[17] Hung H.C., Joshipura K.J., Jiang R., Hu F.B., Hunter D., Smith-Warner S.A., Colditz G.A., Rosner B., Spiegelman D., Willett W.C. (2004). Fruit and vegetable intake and risk of major chronic disease. J. Natl. Cancer Inst..

[18] Thomas D.R. (2006). Vitamins in aging, health, and longevity. Clin. Interv. Aging.

[19] Barker D.J.P., Eriksson J.G., Forsen T., Osmond C. (2002). Fetal origins of adult disease: Strength of effects and biological basis. Int. J. Epidemiol..

[20] McMillen I.C., Robinson J.S. (2005). Developmental origins of the metabolic syndrome: Prediction, plasticity, and programming. Physiol. Rev..

[21] Barlow B.K., Cory-Slechta D.A., Richfield E.K., Thiruchelvam M. (2007). The gestational environment and Parkinson’s disease: Evidence for neurodevelopmental origins of a neurodegenerative disorder. Reprod. Toxicol..

[22] Wu J., Basha M.R., Brock B., Cox D.P., Cardozo-Pelaez F., McPherson C.A., Harry J., Rice D.C., Maloney B., Chen D., Lahiri D.K., Zawia N.H. (2008). Alzheimer’s disease (AD)-like pathology in aged monkeys after infantile exposure to environmental metal lead (Pb): Evidence for a developmental origin and environmental link for AD. J. Neurosci..

